# Is Bitterness Only a Taste? The Expanding Area of Health Benefits of Brassica Vegetables and Potential for Bitter Taste Receptors to Support Health Benefits

**DOI:** 10.3390/nu14071434

**Published:** 2022-03-30

**Authors:** Anqi Zhao, Elizabeth H. Jeffery, Michael J. Miller

**Affiliations:** 1 Division of Nutritional Sciences, University of Illinois, Urbana, IL 61801, USA; anqiz5@illinois.edu; 2 Department of Food Science and Human Nutrition, University of Illinois, Urbana, IL 61801, USA; ejeffery@illinois.edu

**Keywords:** glucosinolate, isothiocyanate, brassica, bitter taste receptor, gut microbiota, nuclear factor-erythroid 2 p45-related factor 2

## Abstract

The list of known health benefits from inclusion of brassica vegetables in the diet is long and growing. Once limited to cancer prevention, a role for brassica in prevention of oxidative stress and anti-inflammation has aided in our understanding that brassica provide far broader benefits. These include prevention and treatment of chronic diseases of aging such as diabetes, neurological deterioration, and heart disease. Although animal and cell culture studies are consistent, clinical studies often show too great a variation to confirm these benefits in humans. In this review, we discuss causes of variation in clinical studies, focusing on the impact of the wide variation across humans in commensal bacterial composition, which potentially result in variations in microbial metabolism of glucosinolates. In addition, as research into host–microbiome interactions develops, a role for bitter-tasting receptors, termed T2Rs, in the gastrointestinal tract and their role in entero-endocrine hormone regulation is developing. Here, we summarize the growing literature on mechanisms of health benefits by brassica-derived isothiocyanates and the potential for extra-oral T2Rs as a novel mechanism that may in part describe the variability in response to brassica among free-living humans, not seen in research animal and cell culture studies.

## 1. Introduction

Brassicaceae (previously termed Cruciferae), known as the mustard family, is composed of a large number of species that are of great economic and nutritional importance. One of the important genera in this family is the genus *Brassica*, which includes vegetables that are consumed worldwide, such as broccoli, kale, brussels sprouts, cabbage, rape, cauliflower, and turnip [1]. Brassica vegetables are a good source of vitamin C, calcium, magnesium, phenolics, tocopherols and carotenoids, as well as dietary fiber [2]. However, a unique group of compounds present in brassica vegetables that is considered to be particularly beneficial to health is the glucosinolates (GSLs), or, more precisely, one group of metabolites of GSLs, the isothiocyanates (ITCs) [3]. It is important to note that intact GSLs are not nearly as bioactive as their ITC metabolites [4,5]. When GSLs are hydrolyzed by either plant β-thioglucosidase or gut microbiota, the ITCs derived from GSLs are highly bioactive compounds. Therefore, the physiological benefits of brassica vegetables are not only dependent on the amount/frequency of consumption, but also on the presence of hydrolyzing enzyme activity [6].

Over the past several decades, a large number of studies focusing on physiological effects of brassica GSLs have been reported, with findings of ITCs’ activities in anti-inflammation, chemoprotection, neuroprotection, anti-obesity, and modulation of gut microbiota, both in clinical studies and in animal/cell culture models [3,7,8]. As a result of rapid development in metagenomic sequencing techniques, the impact of brassica consumption on host microbiota has begun to unfold [9,10,11]. However, in clinical studies, despite numerous animal and cell culture studies suggesting the physiological benefits of brassica and their ITCs, there remains substantial interindividual variation in the extent of conversion of GSL to ITC—and thus in the resulting absorption and bioactivity of ITCs [12]. This variability is sufficiently large that it most frequently disrupts the ability of clinical trials utilizing diverse populations to determine significance for any effects. Here, we discuss recent updates in health benefits of ITCs and factors causing variation in ITC formation that interfere with clinical verification of these benefits, with particular focus on the impact of commensal bacteria on GSL metabolism. In addition, we summarize data supporting the developing idea of a role for ITC in health effects via activation of extra-oral bitter taste receptors (T2Rs).

## 2. Metabolism of Glucosinolates and Resulting ITC Bioavailability: A Key Source of Variability in Health Benefits

### 2.1. Metabolic Fate of Glucosinolates

GSLs are secondary metabolites of brassica with a common structure that comprises a sulfonated oxime moiety, linked with a β-thioglucose group and a variable aglycone side chain derived from one of several amino acids [1,13,14]. The structure of the amino acid precursor determines the classification of GSLs: (1) aliphatic (i.e., glucoraphanin, sinigrin), from Met, Ala, Leu, Ile, and Val; (2) aromatic (i.e., gluconasturtiin), from Phe and Tyr; (3) indolic (i.e., glucobrassicin), from Trp [2]. The content and types of GSL in brassica vegetables are affected by species, cultivation conditions, location in the plant, as well as the post-harvest treatment and cooking conditions [13,14,15]. Although more than 130 GSLs have been identified in plants, glucoraphanin (GRP), sinigrin, gluconasturin, and glucobrassicin are the main GSLs in commonly consumed brassica vegetables [1].

In the whole plant, GSLs are relatively stable because the β-thioglucoside glucohydrolase, called myrosinase, is stored in myrosin granules, away from the GSLs. When there is tissue damage (i.e., cutting, chewing, insects biting), myrosinase gains access to and hydrolyzes GSLs, removing glucose to form an unstable intermediate that rearranges to form an ITC, indole-3-carbinol, nitrile, or epithionitrile, depending on the GSL side chain, pH, the presence of ferrous ions, and the presence of different specifier proteins [16,17]. Neutral pH favors the formation of ITCs, whereas an acidic pH favors the formation of nitriles. The formation of a nitrile is also favored by the presence of ferrous ions and any of a group of plant specifier proteins called epithiospecifier protein (ESP), thiocyanate forming protein (TFP), and nitrile specifier protein (NSP) [18]. If the side chain has a β-hydroxyl group, the unstable intermediate cyclizes spontaneously to form oxazolidin-2-thione [17]. Epithionitriles are formed from GSLs containing an unsaturated terminal side chain [17]. Different cooking conditions and food matrices (such as heating time and temperature, microwaving time and power level, volume of water added, and cutting size) impact the chemical composition of the food ingested, the conversion of GSLs to ITCs, and thus the extent of bioactivity [15,19,20]. For example, sulforaphane (SF, the ITC metabolite of GRP) production from broccoli sprouts is significantly increased by preheating the sprouts to 60 °C for 10 min, destroying the very heat-sensitive ESP (the plant protein that favors the formation of nitriles over ITCs during hydrolysis). However, it then significantly decreases when broccoli sprouts are further heated above 70 °C and up to 100 °C, temperatures that destroy myrosinase [20].

When plant myrosinase is denatured by heating, both gut microbiota and supplementation of exogenous myrosinase have been shown to enhance the conversion of GSLs to ITCs [21,22]. The variation in individuals’ gut microbiomes then plays an important role in the variation seen in the extent of conversion of GSLs to active ITCs in the gut.

**Figure 1:**
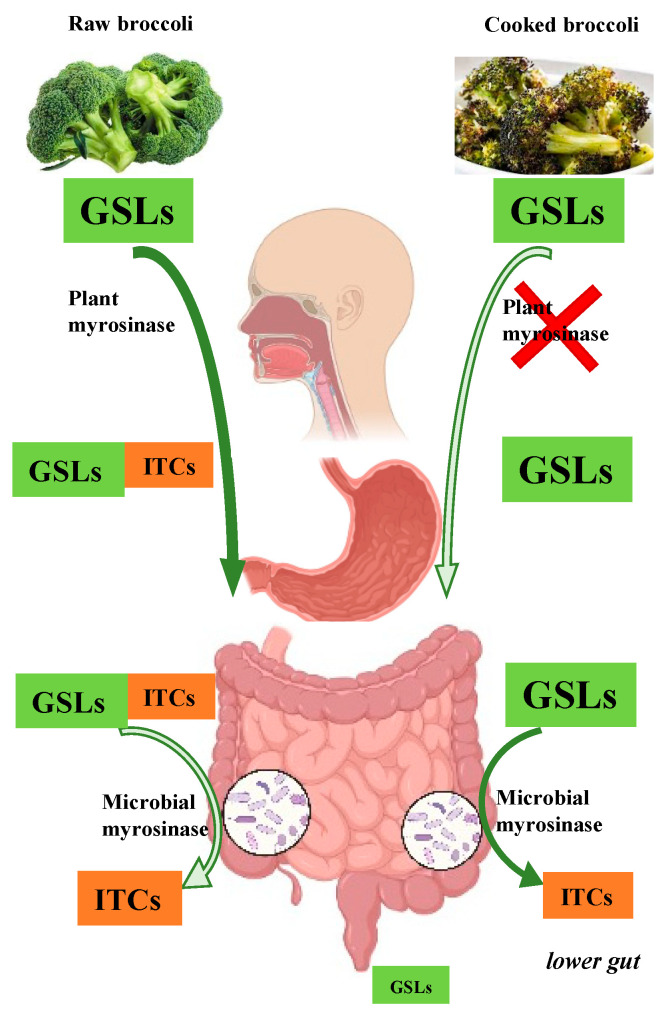
Metabolic fate of glucosinolates in raw vs. cooked broccoli. GSLs, glucosinolates; ITCs, isothiocyanates. Figure created with BioRender.com.

Although a small amount of GSL (i.e., 5% of an oral dose of GRP) may be absorbed intact, most GSLs are hydrolyzed without absorption by the plant myrosinase and/or gut microbiota (Figure 1). The metabolism of GSLs to ITCs often starts in the mouth, where raw brassica vegetables are chewed and GSL is cleaved by myrosinase to release ITC. Studies investigating metabolism of brassica have mainly focused on GRP and its ITC metabolite SF [6,23,24,25]. Once converted from GRP, SF is absorbed by the enterocyte, most likely through passive diffusion [6,26] and then is rapidly conjugated to glutathione inside the cell. This conjugate then enters the circulation, travelling to the liver where it is further metabolized through the mercapturic acid pathway to form SF conjugates of cysteinylglycine (CysGly), and cysteine (Cys). A further metabolite, N-Acetylcysteine (NAC), is formed in the kidney. Any hepatic conjugate may be excreted back into the gut lumen via multidrug resistance protein 2 (MRP2), for passage through the bile duct to the gut. This is followed by reabsorption (termed enterohepatic circulation) or fecal excretion [6,11,23,27]. GRP metabolites in urine are mainly NAC (least reactive, most water-soluble metabolite), with some free SF, SF-Cys, and SF-CysGly [6].

### 2.2. Microbial Metabolism of Glucosinolates

When plant myrosinase is inactivated by cooking brassica vegetables, intact GSLs travel to the lower gut, where gut microbiota with myrosinase-like activities hydrolyze them to bioactive ITCs [17,28]. Compared to the rapid absorption and excretion of ITCs after eating raw brassica, the absorption and excretion of ITCs is both diminished and delayed when cooked brassica are eaten, since there is a significant time delay before GSLs get to the lower gut and undergo hydrolysis by the gut microbiota (Figure 1). In clinical studies, NAC conjugate excretion was found to peak between 3 and 6 h after eating raw cabbage, compared with a peak between 9 and 12 h after eating cooked cabbage [29].

The microbial transformation of GSLs is of great interest since this supports formation of ITCs from brassica consumption, regardless of the cooking method employed, and thus provides greater possibilities for the therapeutic effect of brassica diets [21]. Interestingly, in a dextran sulfate sodium (DSS) mouse model of inflammatory bowel disease (IBD), it was found that both raw broccoli (with active plant myrosinase) and lightly cooked broccoli (plant myrosinase greatly inactivated) were equally effective in protecting the host from DSS-induced colitis, for most of the endpoints, including the clinical score for colitis (disease activity index, DAI), colon length, and lesion severity, with exceptions in gut barrier integrity and proinflammatory markers’ mRNA expression [30]. Compared to raw broccoli, where SF is rapidly released in the mouth and absorbed in the stomach, SF in cooked broccoli is slowly released in the lower gut. Although it is unknown how much of the SF released in the lower gut was due to the remaining myrosinase activity in the partially cooked broccoli, it is possible that SF released from cooked broccoli in the colon by microbial transformation, and some active plant myrosinase directly ameliorated the colitis, even though the total dose of SF may be less than that from raw broccoli [30]. Future studies should evaluate the health benefits of alternate brassica vegetable preparation in conditions that only partially inactivate myrosinase activity and thus impact the location and source of ITC production.

Individual bacterial species capable of metabolizing GSL have been extensively reviewed and found to spread across diverse bacterial phyla, including members of *Firmicutes*, *Bacteroidetes*, *Actinomycetes*, and *Proteobacteria* [10,11]. They include both Gram-positive and Gram-negative, as well as commensal and pathogenic bacteria [10]. Among these, lactobacilli and bifidobacteria have drawn much research interest for another reason: their widely known probiotic properties. Their potential as probiotics has caused the dietary supplement industry to market these bacteria, which may have the serendipitous effect of improving GSL hydrolysis. Further study is needed in this area. Little is known regarding bacterial myrosinase sequences or mechanism of activity. To date, the only known bacterial myrosinase was isolated from a *Citrobacter* strain (WYE1) from soil [31]. A recent study reveals an operon, BT2160-BT2156, within a prominent human gut microbial species—*Bacteroides thetaiotaomicron*—that appears to be required for hydrolysis of GSLs, providing possible insight into an underlying mechanism of gut bacterial hydrolysis of GSLs [32]. Other aspects of bacterial metabolism of GSLs, such as the location of the microbial myrosinase (i.e., intra- or extracellular) or how the bacteria manage the antimicrobial properties of ITCs, are also little understood and need further study.

Emerging animal and clinical studies have focused on understanding the impact of brassica consumption on the composition of the host gut microbiota and their metabolic function, as well as host health in general [21,33,34]. The response of the commensal microbial community to a brassica vegetable diet has been shown to vary [33]. A few studies have shown consistent changes in bacterial composition with brassica consumption. In rats, *Akkermansia manuciphila* was consistently found to be increased by brassica vegetables [11,21,35]. However, more commonly seen across studies are variations in bacterial composition (at the family and genus levels) after brassica consumption in human and animal studies [11]. Quite varied responses of human commensal microbiota to dietary brassica have been found among hosts, which suggests that the host species/environment, and the basal microbiota structure may affect results. In clinical studies, highly variable conversion rates of GSLs to ITCs have been reported, ranging from 1% to 40%, following identical oral administration of a broccoli sprouts extract [36], suggesting that prior individual differences in gut microbial communities lead to variation in GSL bioactivation rates [9,11].

### 2.3. Alternative Pathways of Microbial Metabolism of Glucosinolates

**Figure 2:**
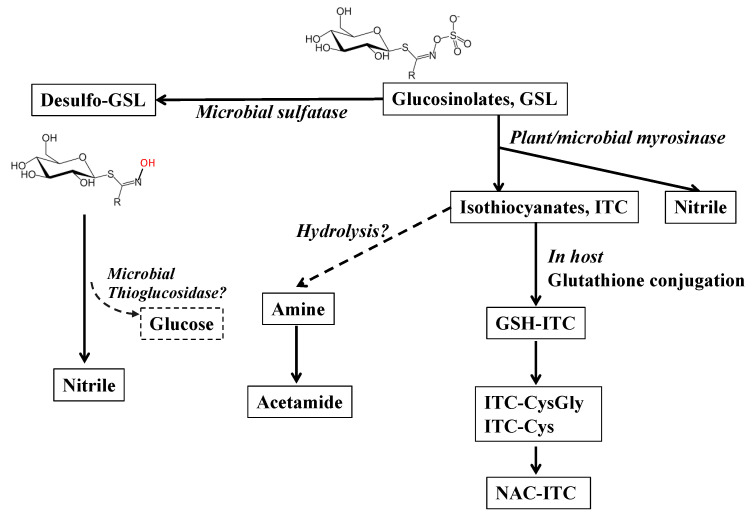
Alternative pathways of microbial metabolism of glucosinolates (GSL). Plant/microbial myrosinase hydrolyzes GSL to form isothiocyanates (ITC) and/or nitrile. ITCs can then be conjugated with glutathione (GSH-ITC) in the host. This conjugate then enters the circulation, travelling to the liver where it is further metabolized through the mercapturic acid pathway to form ITC conjugates of cysteinylglycine (CysGly), and cysteine (Cys). A further metabolite, N-Acetylcysteine (NAC), is formed in the kidney [6]. Alternative pathways of ITCs were reported in flea beetle that ITCs can be hydrolyzed to form amine and acetamide [38,39]. An alternative pathway of GSL metabolism is to form desulfo-GSL by microbial sulfatase [38,39], possibly followed by removal of the glucose by microbial thioglucosidase, and result in a nitrile [44]. Dashed arrows are hypothesized pathways.

Although in vivo and in vitro assessment of GSL metabolites have identified many bacteria with myrosinase-like activities that support formation of ITCs, the transformation rates found to date are relatively low [37]. Recent studies with insect herbivores that eat a variety of brassica (brassica specialists) have identified several additional metabolic pathways for GSLs (Figure 2) [38,39]. As ITCs can be toxic at high concentrations, the gut microbiota of these insects have evolved to avoid ITC accumulation. For example, one mechanism is to further degrade ITCs into nontoxic metabolites. The gut microbiota of cabbage root fly larvae has been shown to hydrolyze ITCs into an amine and a carbonyl sulfide (Figure 2) [40,41]. The amine is then used as a nitrogen source for the microorganism. Another mechanism is the removal of the sulfate group on GSLs by a sulfatase [42,43]. As the sulfate group is essential for the rearrangement that occurs after myrosinase reacts with GSLs, sulfate removal results in disruption of ITC production, leading to nontoxic desulfo-GSLs that cannot break down to ITCs. Furthermore, the desulfo-GSLs could provide a valuable glucose molecule to gut microbes that exhibit sulfatase and thiohydrolase activity, so that GSLs become an energy source, without toxicity from ITCs (Figure 2). There are several studies showing human gut bacteria with sulfatase activity that form desulfo-GSL in vitro [44,45].

The dynamic interactions between brassica vegetables and the gut microbiota still lack basic mechanistic knowledge. It is unclear whether the increased efficiency in GSLs’ transformation to ITCs seen following prolonged exposure to brassica diets [21] also leads to increased microbial breakdown of ITCs to nontoxic metabolites. It is important to note that all of these pathways may occur concurrently within the gut microbiota, yet little is known about how brassica consumption may impact these pathways or how these competing pathways may explain the individual variation in the conversion of GSLs to ITCs seen in clinical studies.

## 3. Health Benefits of Brassica Vegetables and Associated Mechanisms

ITCs derived from brassica GSLs exert a wide range of physiological effects that impact the host’s immune and metabolic systems. Numerous studies have reported antioxidant and anti-inflammatory activities of ITCs, mainly through inducing nuclear factor-erythroid 2 p45-related factor 2 (Nrf2) and suppressing nuclear factor-κB (NF-κB) pathways [46]. SF, among all the ITCs, is best-known for its chemopreventive effects [7]. Other ITCs, such as benzyl isothiocyanate (BITC) and phenethyl isothiocyanate (PEITC), have anticancer effects, similarly impacting the different stages of cancer development [47]. Isothiocyanates have been found to ameliorate several signs of metabolic syndrome, including diabetes [48], which may be due to ITC-dependent antioxidant and anti-inflammatory activities. In addition, several studies investigating the antimicrobial effects of ITCs, have suggested a potential role in antipathogenic infection/inflammation in the host [49]. In the following paragraphs, we will summarize known mechanisms underlying these health effects. In addition, we will discuss the recent identifications of bitter taste receptors (T2Rs) in the gut, and their potential impact on host glucose homeostasis, satiety, and more, such as novel mechanisms by which brassica may provide additional health benefits.

### 3.1. Health Benefits of ITCs Working via Nrf2 Pathways

Nrf2 is the most-studied and understood target of ITCs, particularly of SF. It is a key transcription factor that is expressed in many organs, including the liver, kidney, lung, and digestive tract [50]. Kelch-like ECH-associated protein1 (Keap1) regulates the activity of Nrf2, acting as a repressor protein. Upon exposure to electrophiles and/or reactive oxygen species (ROS), Nrf2 dissociates from Keap1 and translocates to the nucleus, where it binds DNA at the antioxidant/electrophile response elements (ARE/EpRE), which then upregulates the expression of phase II detoxifying enzymes and antioxidant proteins, including glutathione S-transferases (GST) and NAD(P)H:quinone oxidoreductase-1 (NQO1) [51,52]. The regulation of Nrf2 by Keap1 and subsequent Nrf2 pathways following the destruction of Keap1 has been reviewed [53]. Besides regulating the expression of antioxidative proteins/enzymes, activation of the Nrf2/ARE system also plays an important role in inhibiting the overproduction of proinflammatory cytokines, such as IL-6, TNFα, and Il-1β [53], mainly through suppressing the NF-κB pathway [53,54]. In addition to antioxidant and anti-inflammatory effects, these Nrf2-dependent activities relate to the physiological actions of Nrf2 in cytoprotection, chemoprevention, and in preventing a number of chronic metabolic diseases, including type 2 diabetes [55]. Furthermore, chemoprotection by Nrf2 includes triggering cell apoptosis and inducing epigenetic alteration [51]. In total, there are about 200 genes recognized to be regulated by Nrf2, including genes involved in cytoprotection, gene transcription, and glucose and lipid metabolism [51,54].

#### 3.1.1. Prevention of Oxidative Stress and Inflammation

The major impact of the interaction of ITCs with the Nrf2/NF-κB pathway(s) is the control of oxidative stress and chronic inflammation [48]. Many studies have reported that purified ITCs (including SF, allyl isothiocyanate (AITC), BITC, PEITC) or brassica diets increase Nrf2-dependent antioxidant gene expression (NQO1, superoxide dismutase (SOD), heme oxygenase-1(HO-1), and others) and/or reduce proinflammatory mediators’ gene expression (NF-κB, IL-6, cyclooxygenase 2 (COX2), vascular cell adhesion molecule 1 (VCAM-1)) in cell and animal models of cancer or inflammation [30,56,57,58]. ITC-induced bioactivities reported using cell culture and animal studies typically show consistent results, since these models’ potential variables, including food and growing conditions, are precisely controlled. However, it is exactly the variability in free-living humans that makes experimental results less translatable to ITC actions in humans/clinical trials.

#### 3.1.2. Chemopreventive Effects

Among the key stages of cancer development (initiation, promotion, progression), ITCs are found to inhibit initiation and delay progression [59,60]. By modulating the activities of phase I (i.e., cytochrome P450) and phase II detoxifying enzymes (e.g., glutathione transferase), ITCs lessen the exposure of cells to environmental carcinogens and defend against oxidative damage [61]. Progression may be interrupted by triggering cell apoptosis, autophagy, and induction of epigenetic alteration. Recent reviews have focused on anticancer effects of SF, summarizing updates in knowledge of mechanism [7,46].

#### 3.1.3. Metabolic Syndrome

Prolonged low-grade inflammation is associated with the development of chronic diseases such as metabolic syndrome. Metabolic syndrome is characterized as a group of risk factors specific for cardiovascular disease, including obesity, diabetes, and heart disease. The anti-inflammatory activities of ITCs contribute to their impact on prevention of metabolic syndrome. The impact of the ITC-Nrf2 pathway on metabolic syndrome has been reviewed [48,62]. In a recent study using Nrf2 knockout mice, Nagata and colleagues found that dietary GRP (the precursor to SF) attenuated high-fat-diet-induced weight gain, insulin resistance, and hepatic steatosis in wild-type mice but not in Nrf2 knockout mice, suggesting these activities are Nrf2-dependent [55]. Similarly, Axelsson and colleagues found that SF lowered glucose production in H4IIE cells, a rat hepatoma cell line, but knockdown of Nrf2 attenuated the beneficial impact of SF on glucose production, suggesting that glucose reduction by SF is mediated via Nrf2 [63]. To validate their findings in vivo, they studied the effect of SF and an SF-generating diet on glucose homeostasis in mice with diet-induced diabetes and in obese patients with type 2 diabetes (T2DM), respectively.

### 3.2. Health Benefits of ITCs through Mechanisms Other Than Nrf2

The potential for ITCs to act as antimicrobial agents, controlling both pathogenic and commensal bacteria, is evident [49]. AITC, BITC, PEITC, and SF have all been shown to exhibit antimicrobial activities against various pathogenic bacteria isolated from human intestine, mouth, and feces [49,64]. Brassica plants also exert both antimicrobial and pesticidal activity, since when pests damage plant tissue, this releases myrosinase, which causes hydrolysis of GSLs and production of potentially toxic doses of ITC. The mechanisms of ITCs’ antibacterial activities, include damaging membranes, inhibiting quorum sensing, and induction of heat-shock responses, have been reviewed [49].

Both animal and clinical studies have reported that consumption of brassica vegetables can cause alteration in the host gut microbiota, structurally and/or functionally [21,34,35,65,66]. Emerging evidence shows a reciprocal interaction between a number of phytochemicals and host gut microbiota: whereas these phytochemicals modulate gut microbial structure, the gut microbiota also impacts the bioactivity of the phytochemicals by transforming them (here specifically transforming GSL into bioactive ITC). Our own studies found that four days of prefeeding with a diet of 10% cooked broccoli or GRP-rich extract altered rat cecal microbiota function, resulting in significantly increased efficiency in producing bioactive ITCs ex vivo, compared to cecal bacteria from rats fed a control diet [21]. Similar results have been shown in clinical studies, where broccoli consumption altered human gut microbiota and the changes were associated with increased ITCs in plasma and urine [34]. In addition, a randomized crossover study reported that two-week consumption of a high-brassica diet was associated with a reduction in sulphate-reducing bacteria, the group of bacteria that may cause gut inflammation by producing hydrogen sulfide [67]. However, these studies used whole broccoli and thus could not confirm that the observed changes were due solely to the increase in microbially produced ITCs. These data suggest that alteration in the microbiome caused by brassica diets may enhance delivery of health benefits.

## 4. A Novel Interaction: The Bitter Brassica Vegetables and Intestinal Bitter Taste Receptors

Bitter taste receptors (generically known as T2Rs; TAS2R for humans and Tas2r for mice) belong to the superfamily of G protein-coupled receptors (GPCRs). Originally identified in oral taste buds as sensors for bitterness, they allow for warning of potential poisons in foods [68]. Unlike the individual sweet taste receptor and the individual umami taste receptor, there are 25 functional T2Rs identified so far in humans, allowing response to a wide range of bitter-tasting compounds [69]. Over the past decade, scientists have identified these T2Rs in extra-oral systems, including the gastrointestinal tract, the respiratory system, the cardiovascular system, the nervous system, adipose tissue, and the immune system [70,71,72]. Studies have shown that activation of extra-oral T2Rs plays important physiological roles, including regulating appetite and blood glucose response, modulating airway innate immunity, ameliorating obstructive lung diseases, and delivering neuroprotective effects [73,74,75,76,77,78].

**Figure 3:**
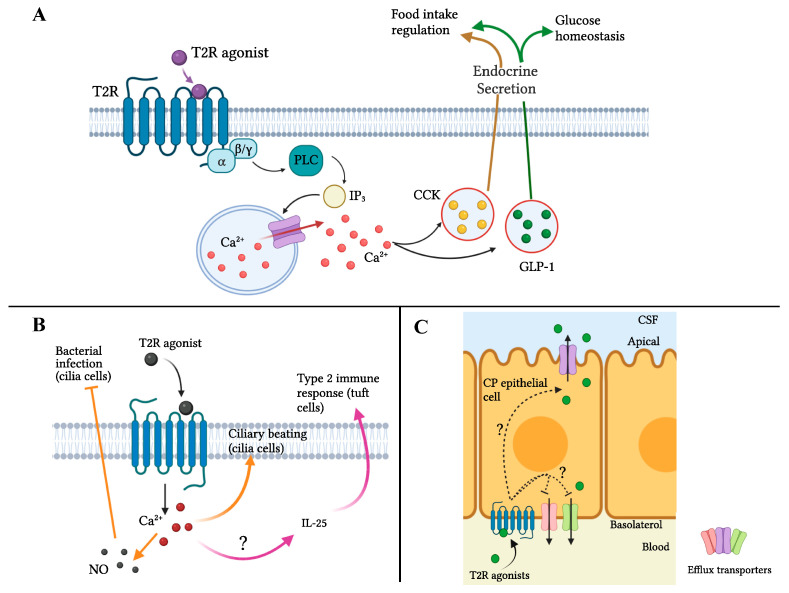
Proposed model of T2R signaling pathways impacting physiological health. (**A**) Glucose metabolism and obesity: in enteroendocrine cells, T2R agonists activate T2Rs to cause increased calcium release from calcium channels on the endoplasmic reticulum, leading to GLP-1 and/or CCK secretion. (**B**) Innate immunity: in cilia cells, the activation of T2Rs by T2R agonists induce calcium flux, leading to increased ciliary beating and/or production of NO; in tuft cells, activation of T2Rs causes a type 2 immune response by inducing secretion of IL-25. (**C**) Neuroprotection: in human CP epithelial cells, T2Rs regulate efflux transporters at the BCSFB may facilitate transportation of neuroprotective molecules, shown to date for resveratrol [78]. Abbreviations: T2R, bitter taste receptor; GLP-1, glucagon-like peptide-1; CCK, cholecystokinin; NO, nitric oxide; CSF, cerebrospinal fluid; CP, choroid plexus; BCSFB, blood–cerebrospinal fluid barrier. Figure 3A,B based on [70,72]. Figures created with BioRender.com.

Several proposed mechanisms, including gut hormone secretion, secretion of antimicrobial molecules, and regulation of ATP-binding cassette (ABC) transporters at the blood–brain barrier (BBB)/blood–cerebrospinal fluid barrier (BCSFB), have linked the activation of extra-oral T2Rs with potential roles in physiological paths (Figure 3). More specifically, mouse studies show that T2R agonists, such as denatonium benzoate (BD), phenylthiocarbamide (PTC), and KDT501, delay gastric emptying, increase satiety, enhance glucose tolerance, and cause weight and fat mass loss through inducing GLP-1 and/or CCK secretion [73,79]. Genetic variations in T2Rs, especially TAS2R38, was found to be associated with increased risk in obesity and dysregulated glucose and insulin in human subjects [80,81]. In one study, the expression of T2Rs in mouse colon tissue was shown to be induced by a high-fat diet, suggesting that dietary T2R agonists may be especially helpful for a population enjoying a high-fat diet [82]. The same research group also found that a fecal transplant from obese mice fed a high-fat diet to mice on a low-fat diet resulted in a 7–11-fold increase in expression of T2Rs in the large intestine and a significant positive correlation between T2R expression and intestinal abundance of *Akkermansia* [82,83]. It is important to note that the expression and abundance of each T2R subtype varies among different tissues and cell types and genetic variant, meaning that to be effective, a specific T2R agonist needs to be presented at the location where the associated specific target T2R is expressed and in sufficient abundance that, upon activation, this leads to a physiological effect.

There is potential that genetic variation with T2Rs can impact their function. For example, genetic differences in the TAS2R38 gene (PAV, functional vs. AVI, nonfunctional) may impact an individual’s health status. These alleles are well-known to impact bitter taste perception from ITCs [84]. Furthermore, Tran and colleagues found that AITC exerts bactericidal and anti-inflammatory actions in human monocytes from PAV/PAV subjects, not AVI/AVI subjects, suggesting that T2R genetic variation may have a broader impact than just oral bitter taste perception [85].

Whereas ITCs play a significant role in Nrf2-dependent bioactivities, a role for ITCs in interacting with extra-oral T2Rs may also offer multiple health benefits. ITCs are believed to be the compounds responsible for the bitter and pungent flavor of brassica vegetables [86]. Evidence suggesting that T2Rs respond to ITCs has been reported by several research groups, potentially due to the N-C=S moiety contained in these compounds [84,87]. One cell culture study challenged 25 human T2Rs with 104 bitter chemicals and determined that sinigrin (SN, the major GSL in cabbage) and AITC (the hydrolysis product of SN) are capable of activating TAS2R16 and/or TAS2R38 [88]. Similarly, SN and AITC activate mouse T2Rs, including Tas2r105 and Tas2r135, respectively. However, it is important to note that the threshold concentration for activating TAS2R38 (i.e., triggering intracellular calcium release) was 10-fold greater for SN (100 µM) than for AITC (10 µM), suggesting that, at physiologically relevant concentrations, AITC is more potent at activating T2R than SN is [88]. On the other hand, this study used HEK293T cells transfected with individual T2Rs, the threshold concentration of AITC/SN for activating the T2Rs may not be translatable to physiological conditions, considering the variation in individual T2Rs’ expression and abundance along the gastrointestinal tract. The same research group also reported that another ITC, PEITC, is an agonist for Tas2r105 [69]. Although shown to be T2R activators, any subsequent physiological impact following T2R activation was not reported. Studies are needed to relate ITC activation of T2Rs to physiological endpoints and to differentiate between Nrf2-dependent and T2R-dependent effects. The possibility of individual variation in the conversion of GSLs to ITCs leading to variations in physiological effects caused by T2Rs also requires further investigation.

### 4.1. Glucose Metabolism and Obesity

Cell culture and animal studies have indicated that bitter-tasting vegetables/food components impact glucose homeostasis, food intake, and weight gain via activation of intestinal T2Rs (Figure 3A) [73,79]. For example, gavage of bitter-tasting compounds, which bypass any impact in the mouth, causes induction of CCK-dependent hindbrain activation, which plays a role in reducing food intake. Similarly, intragastric administration of a bitter mixture increases the release of the hormone ghrelin, whose long-term effect correlates with a slowing of gastric emptying [73,75,89,90]. Kok and colleagues found that a purified bitter derivative from hops ameliorates features of metabolic syndrome in diet-induced obese mice, possibly partly by increasing GLP-1 release via activating intestinal Tas2r108 [79]. Enteroendocrine hormones serve as important mediators in exerting bioactivities of bitter-tasting compounds [72]. Although ITCs have been shown to be T2R activators, the activation by ITCs of intestinal T2Rs leading to enteroendocrine hormone secretion and glucose regulation is still unclear. A recent randomized control trial reported that bitter and strong-tasting brassica vegetables have a significant impact on insulin sensitivity, fasting glucose levels, body fat mass, and blood pressure, compared to a nonbitter control diet [91]. It has been hypothesized that these effects could possibly be Nrf2-dependent [62], but this requires further study. A correlation between T2R binding and beneficial impacts on glucose metabolism is insufficient to show causation. Further studies are needed to prove a mechanistic relationship.

### 4.2. Innate Immunity

Activation of T2Rs in the respiratory and gastrointestinal tract has been suggested to be a defensive mechanism of these organs in response to external infections, such as bacteria, viruses, fungi, and their derived products [72]. The role of bitter-tasting compounds in the respiratory immune system has been the focus of most of the studies in this area (reviewed by Lu and colleagues [70]). It has been reported that activation of T2Rs in human motile cilia increases the frequency of cilial beating and therefore improves the clearance of mucus which might contain noxious substances (i.e., microbes and microbial derivatives) [92]. In addition, T2Rs in the respiratory tract act as sensors for the quorum-sensing molecules produced by microbes, which induce a series of antimicrobial actions, such as generating nitric oxide (NO) that slows down bacterial multiplication (Figure 3B) [70,93]. Moreover, it has been proposed that activation of T2Rs in mouse tuft cells may initiate type 2 immunity against parasites by inducing secretion of IL-25 and a subsequent immune response, leading to increased hyperplasia of tuft cells and goblet cells (Figure 3B) [70,94]. Although brassica consumption leads to release of ITCs in the gut, whether ITCs have any effect on these immune cells has not been reported—or even whether ITCs interact with T2Rs expressed in tuft cells. AITC was reported to stimulate NO production and exert bactericidal activity in human monocytes through activating T2R38 [85]. However, ITCs’ role in improving activity of the innate immune system in vivo remains to be explored. As the authors suggested, in future studies, genetic variants might be useful tools to ask some of these questions.

### 4.3. Neuroprotection

Several bitter-tasting phytochemicals from a variety of fruits and vegetables have been tested for protective action against degenerative nerve diseases, using both cell culture and animal models (reviewed by Duarte and colleagues) [95]. However, whether the neuroprotective actions of these bitter compounds are mediated by T2Rs is unknown. One study showed that T2R14 expressed on epithelial cells of the human choroid plexus can be activated by the bitter ligand resveratrol. Once activated, these T2R can trigger regulation of the efflux transporters at the junction between the epithelial cells of the choroid plexus (termed the blood–cerebrospinal fluid barrier) to facilitate transportation of resveratrol into cerebrospinal fluid (Figure 3C) [78]. Using a cell culture system, two ITCs, SF and AITC, have been found to exert “neuroprotective actions”; the possible underlying mechanisms, such as the Nrf2/ARE pathway, have been reviewed by Klomparens and Ding [96,97,98]. Although functional T2Rs are expressed in the human and rodent nervous systems, including T2R38, which is known to be an AITC target, direct evidence suggesting a role for T2Rs is limited to cell culture at this time [71,95,99]. Although the anti-inflammatory activities of T2Rs in the central nervous system (CNS) are less studied than in other systems such as the respiratory system, it is feasible to hypothesize that activation of T2Rs inhibits neuroinflammation, potentially by T2Rs that sense pathogenic/toxicogenic molecules in the brain, as they do in the respiratory system [95]. It has been demonstrated that SF and AITC can cross the blood–brain barrier and accumulate in the CNS [98,100]. If able to interact with T2Rs expressed in neurons, ITCs may have a therapeutic potential to counter neuroinflammation and neurotoxicity. Further studies are required to investigate the potential role of ITCs interacting with T2Rs in neuroprotection.

## 5. Conclusions and Future Perspectives

In conclusion, brassica vegetables are popular and consumed worldwide. Similarly, their health benefits are widely studied, frequently leading to novel benefits being revealed. However, interindividual variations in conversion of GSLs to ITCs and variations in an individual’s response to ITCs could adversely influence proof of any potential health benefits. One of the major tasks for improving accessibility to these health benefits is therefore to improve ITCs’ bioavailability. As we discussed in the previous paragraphs, the source factors leading to variations in ITCs’ bioavailability and bioactivity could include (1) processing/cooking method for brassica vegetables; (2) the amount and frequency of brassica vegetable consumption; (3) the host microbiota composition and activity; (4) the host genotype, such as functional T2R38 vs. nonfunctional T2R38. Understanding the impact of brassica consumption on different microbial GSLs/ITCs metabolic pathways may help with explaining these variations and improve ITC’s physiological benefits. Additionally, besides the most studied Nrf2/ARE system as the underlying mechanism of action of ITCs, here we propose that recent findings of ITC–T2R interaction at extra-oral T2Rs support the possibility of a novel additional pathway for exerting physiological effects by brassica vegetables. However, this is presently only a hypothesis based on current evidence until more research is carried out.

More studies are needed to determine the interaction between ITCs and T2Rs, in multiple organs/tissues. For example, it would be interesting to identify the specific T2R(s) target(s) of different ITCs. Since any given T2R may be activated by multiple ligands or only by a single ligand, our working hypothesis is that each ITC may have their primary T2R target, interacting with others at increased doses, or at greater T2R concentrations. Given the fact that expression of individual T2Rs varies among organs/tissues, evaluating the relative expression levels of individual T2R along the gastrointestinal tract and identifying each ITC’s target T2R(s) will be important for further exploring ITC’s physiological impacts. Moreover, using T2R(s) knockout/knockdown models to evaluate the impacts will be useful to establish the causal relationship and differentiate between T2R-dependent and Nrf2-dependent effects. Studies are needed to compare ITCs to other plant bitter compounds such as triterpenoids or whether T2Rs are involved in the acquisition of bitter taste acceptance. In addition, it is reported that genetic differences in the T2R38 gene (PAV, functional vs. AVI, nonfunctional) may impact an individual’s health status. For example, single nucleotide polymorphisms (SNPs) in T2Rs have been reported to be associated with dysregulated glucose and insulin homeostasis by evaluating 145 subjects with T2DM and 358 control subjects [81]. Moreover, PAV/PAV monocytes but not AVI/AVI monocytes respond to AITC, suggesting that AITC modulates immune responses in a functional T2R-dependent manner [85]. It would also be interesting to evaluate the physiological impact of the interaction between T2Rs and ITCs in clinical studies in subjects with different microbiota background and/or T2R genotype. These data and strategies provided new scope for evaluating ITCs’ physiological impact in the context of personalized nutrition, such as determining the benefits of consuming brassica based on an individual’s bitter taste perception, which warrants further investigation.

## Data Availability

Not applicable.
